# Exosome Biogenesis in the Protozoa Parasite *Giardia lamblia*: A Model of Reduced Interorganellar Crosstalk

**DOI:** 10.3390/cells8121600

**Published:** 2019-12-09

**Authors:** Sofía Moyano, Juliana Musso, Constanza Feliziani, Nahuel Zamponi, Lorena Soledad Frontera, Andrea Silvana Ropolo, Adriana Lanfredi-Rangel, Marco Lalle, María C. Touz

**Affiliations:** 1 Instituto de Investigación Médica Mercedes y Martín Ferreyra, INIMEC–CONICET–Universidad Nacional de Córdoba, Friuli, Córdoba 2434, Argentina; smoyano@immf.uncor.edu (S.M.); jmusso@immf.uncor.edu (J.M.); cfeliziani@immf.uncor.edu (C.F.); zamponi.n@gmail.com (N.Z.); lfrontera@immf.uncor.edu (L.S.F.);; 2 Serviço de Microscopia Eletrônica, Centro de Pesquisas Gonçalo Moniz, FIOCRUZ-BA, Salvador 40296-710, Brazil; alrangel@bahia.fiocruz.br; 3 Department of Infectious Diseases, Foodborne and Neglected Diseases Unit, European Reference Laboratory for Parasites, Istituto Superiore di Sanità, viale Regina Elena 299, 00161 Rome, Italy; marco.lalle@iss.it

**Keywords:** protozoa, exosome, organelle crosstalk, ESCRT complex

## Abstract

Extracellular vesicles (EVs) facilitate intercellular communication and are considered a promising therapeutic tool for the treatment of infectious diseases. These vesicles involve microvesicles (MVs) and exosomes and selectively transfer proteins, lipids, mRNAs, and microRNAs from one cell to another. While MVs are formed by extrusion of the plasma membrane, exosomes are a population of vesicles of endosomal origin that are stored inside the multivesicular bodies (MVBs) as intraluminal vesicles (ILVs) and are released when the MVBs fuse with the plasma membrane. Biogenesis of exosomes may be driven by the endosomal sorting complex required for transport (ESCRT) machinery or may be ESCRT independent, and it is still debated whether these are entirely separate pathways. In this manuscript, we report that the protozoan parasite, *Giardia lamblia*, although lacking a classical endo-lysosomal pathway, is able to produce and release exosome-like vesicles (ElV). By using a combination of biochemical and cell biology analyses, we found that the ElVs have the same size, shape, and protein and lipid composition as exosomes described for other eukaryotic cells. Moreover, we established that some endosome/lysosome peripheral vacuoles (PVs) contain ILV during the stationary phase. Our results indicate that ILV formation and ElV release depend on the ESCRT-associated AAA+-ATPase Vps4a, Rab11, and ceramide in this parasite. Interestingly, EIV biogenesis and release seems to occur in *Giardia* despite the fact that this parasite has lost most of the ESCRT machinery components during evolution and is unable to produce ceramide de novo. The differences in protozoa parasite EV composition, origin, and release may reveal functional and structural properties of EVs and, thus, may provide information on cell-to-cell communication and on survival mechanisms.

## 1. Introduction

*Giardia lamblia* (syn. *G. duodenalis; G. intestinalis*) is a globally distributed protozoan parasite that can inhabit the small intestine of mammals including humans, causing a diarrheal disease known as giardiasis. Ingestion of *G. lamblia* cysts present in contaminated water or food or following contact with feces of infected hosts are the common routes of infection [1]. Human giardiasis is the most common cause of diarrheal disease not associated with viruses or bacteria and can affect people with normal or altered immune systems [1]. Inside the host, the cysts release trophozoites that, following active replication, colonize the upper part of the small intestine and cause the symptoms. *G. lamblia* is an extracellular parasite that adheres to the apical surface of intestinal epithelial cells (IEC) and, by coating the host intestine, impairs the absorption of nutrients and micronutrients, thus leading to weight loss and ultimately to malnutrition, especially in children living in highly endemic areas [2]. The pathogenic effect of trophozoites has been also associated with the secretion of several proteins [3,4] and extracellular vesicles (EVs) [5] that contribute to cellular damage to the IEC and counteract the host’s immune response.

Although several studies have focused on characterizing the *Giardia* secretome, it was only recently that the existence and the role of EVs in *Giardia*–host interaction emerged as a crucial research topic. EVs are a heterogeneous population of secreted membrane vesicles with different biogenesis routes, biophysical properties, and functions both in physiological conditions and diseases [6,7]. The release of EVs is a generalized biological process, which is conserved through different species, representing an important mode of intercellular communication and serving as a vehicle for the transfer of cytosolic and membrane proteins, lipids, and nucleotides [6]. EVs may have an endosomal origin or be formed from the plasma membrane: those that are formed from multivesicular bodies (MVBs) are intraluminal vesicles (ILVs) (termed exosomes when released into the extracellular milieu), while those that directly bud from the plasma membrane are microvesicles (MVs) [7].

The sorting of exosomal material is still under study, and the analysis of exosome composition has highlighted some heterogeneity in extracellular vesicles [8]. However, recent studies have shown that the budding of ILVs (and thus exosome formation) depends on at least three independent and cell-specific processes: (i) the participation of the ESCRT machinery [9], (ii) a ceramide-based mechanism [10], and (iii) a tetraspanin-sorting machinery [11,12].

**Figure 1:**
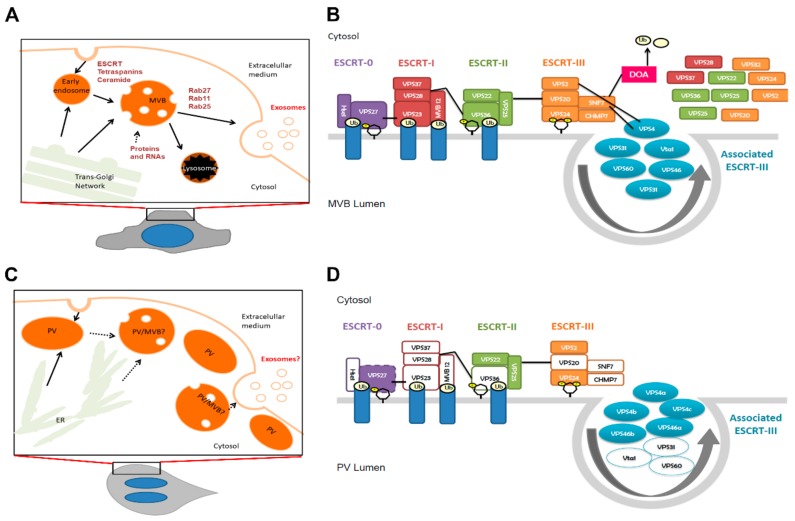
Exosome biogenesis: (**A**) In mammalian cells, exosomes originate from the multivesicular bodies (MVBs) in the endosomal pathway. Formation of the intraluminal vesicles (ILVs) of MVBs seems to require endosomal sorting complex required for transport (ESCRT) proteins, tetraspanins, and ceramide, while Rab proteins were shown to be involved in exosome secretion (Adapted from Reference [27]). (**B**) The ESCRT assembly occurs by the association of the protein subcomplexes to phosphoinositides and ubiquitylated cargoes (Ub) and with each other, sequentially. Later in the pathway, ubiquitin is removed by a de-ubiquitinase (degradation of alpha-4-Doa4-) via ESCRT-III. Disassembly of the complexes is orchestrated by the AAA+-ATPase Vps4. (**C**) In *Giardia* trophozoites, exosomes seem to originate in the peripheral vacuoles (PVs) but the molecules and the related organelles involved in exosome biogenesis are still undefined. (**D**) *Giardia* lacks the whole set of ESCRT components, and those identified in *Giardia* are reported in full rectangles: ESCRT-0 in magenta, ESCRT-I in red, ESCRT-II in green, and ESCRT-III in orange. ESCRT-III-associated proteins are in ellipse, and those identified in *Giardia* are in full-light blue.

The most studied molecular mechanism involved in the biogenesis of MVB and ILV en route to the formation of exosomes is the mechanism mediated by ESCRTs, well-conserved from Archaea to animals (Figure 1A) [13,14]. ESCRTs are cytosolic proteins which sequentially assemble into four multiprotein complexes, recruited to the membrane of early endosomes. The currently accepted model for the distribution and function of each component of the ESCRT machinery during the formation of ILVs within the MVBs is shown in Figure 1B. The four ESCRT complexes are recruited to endosomes via their interaction with membranes, clathrin, and ubiquitin (Ub) and with each other. The recognition of phosphatidylinositol-3 phosphate (PI3P) by the FYVE zinc finger domain of Vps27 (ESCRT-0) and by the GRAM-Like Ubiquitin-binding in EAP45 (GLUE) domain of Vps36 (ESCRT-II) or of phosphatidylinositol 3,5-bisphosphate (PI3,5P_2_) by Vps24 (ESCRT-III) may contribute to early or late endosomal localization of the components. All the complexes except ESCRT-III are able to recognize and bind ubiquitinated cargo. ESCRT-III orchestrates the last steps in the process in which Ub is removed by a deubiquitinase and the complexes are dismantled by the AAA+-ATPase Vps4. Budding is promoted by a curvature induction factor that may flex the membrane when it is located in the neck of the budding ILV. Did2, Vps2, and Vps60 have been shown to interact with the Vps4–Vta1 complex [15,16].

In contrast to the interconnected network that involves early/recycled/late endosomes, MVBs, and lysosomes/vacuoles, *G. lamblia* possesses peripheral vacuoles (PVs), which comprise a tubular/vacuolar network polarized below the plasma membrane, functioning at the same time as endosomes and lysosomes [17,18] (Figure 1C). Although the presence of ILVs inside the PVs has been reported [5,19,20,21], it was not addressed whether the ESCRT machinery is involved. In fact, *Giardia* harbors a reduced ESCRT machinery with only putative orthologs for the Vps22 and Vps25 (ESCRT-II), the Vps2 and Vps24 (ESCRT-III), and the Vps46(a-b) and the AAA-ATPase Vps4(a–c) identified in its genome [22,23]. The same group had identified a Vps27 putative protein that contains the FYVE domain, which preferentially binds PI3P and of which the expression showed a selective localization in endosomes enriched in PI3P in *S. cerevisiae* [24] (Figure 1D).

Another known mechanism involved in ILV and exosome formation relies on ceramide production by neutral sphingomyelinase 2, generating endosomal membrane deformations that bud ILVs in vitro [10]. However, ceramide is not synthesized de novo by *G. lamblia*, [25] but is taken up from the extracellular milieu via the clathrin-mediated endocytic pathway and targeted to the endoplasmic reticulum (ER)/perinuclear membranes [26]. Moreover, there is a presumption that sphingomyelinase plays an important role during *Giardia* differentiation into cysts, increasing the pool of ceramide by degrading intestinal and cellular sphingomyelin to generate excess ceramide [25].

Exosomes are rich in tetraspanins, transmembrane proteins that interact with a large variety of signaling proteins [28]. The direct role of tetraspanins in exosome formation was indicated in studies performed in tetraspanin-deficient mice and in shRNA knockdown cells, which describe defects in exosome secretion [29,30,31]. Contrariwise to other eukaryotic cells, no tetraspanin orthologs are present in *Giardia*, but we cannot exclude the participation of other membrane proteins in the generation of ILVs and exosomes in this parasite.

The Rab family of small GTPases has been reported to contribute to exosome and microvesicle formation [9]. In particular, eight Rabs have been associated with exosome secretion in *C. elegans*, and five of them (Rab2, Rab7, Rab11, Rab27, and Rab35) have a role in EV biogenesis in other organisms [32,33]. However, the specific role of these Rabs in exosome release is yet to be defined. Instead of the more than 60 members of the Rab family identified in mammalian cells, *G. lamblia* has only three predicted Rab proteins, solidly grouped with orthologs from other eukaryotes in phylogenetic analyses: Rab1; Rab2a/b, related to exocytic vesicular trafficking [34]; and Rab11, associated with cytokinesis [35,36] and differentiation [37,38]. In human cells, Rab11 is required for MVB tethering to the plasma membrane and exosome release [38,39], while in *C. elegans* and *Drosophila*, Rab11 is involved in MV budding as well as exosome release [32,40,41,42].

In this work, we report on the presence of exosome-like vesicles (ElV) in the in vitro culture of *G. lamblia* trophozoites. These ElVs are comparable in size, morphology, density, and protein and lipid markers to exosomes from other species. Also, we found that the ESCRT-associated protein Vps4a, Rab11, and ceramide play a significant role in the generation of ILVs inside the PVs and the production of ElV in *G. lamblia*.

## 2. Materials and Methods 

### 2.1. GlVps4a and Rab11 Expression

The *glvps4a* open reading frame was amplified from genomic DNA using the sense f1 oligonucleotide (5′-CATT**CCATGG**CCATTGTTCCTGGTCGAAACATTG-3′) and antisense r1 oligonucleotide (5′-CATTGTATACGGTGGAGCCGAACTCCGCTGTGAACTT3′) (with the *Nco*I site in boldface and the *Bstz*171 site underlined) and cloned into the plasmid pTubH7-HApac [43] to generate the pTubVps4a-HApac episomal vector. To generate the ATP hydrolysis-deficient Vps4 mutant, the *glvps4a* gene was mutated in the E228 to Q228 by site mutation using the Site-Directed Mutagenesis QuikChange II Kit (Aginent), the pTubVps4a-HApac vector, the sense f2 (5′-GGCGGGCAGACTGGCCGACGTACTT-3′) and the antisense r2 (5′-AAGTACGTCGGCCAGTCTGCCCGCC-3′) overlapping template oligonucleotides, following the protocol described by the company (Agilent, Santa Clara, CA, USA). The generated pTubVps4a_E228Q_-HApac vector was used for trophozoite transfection. To express GlRab11-HA, the *glrab11* open reading frame was amplified from genomic DNA using the sense f3 oligonucleotide (CAAT**GGGCCC**ACTGACGCGTACGACCATCTTTA) and antisense r3 oligonucleotide (CAATCCCGGGGCAACGCTTCTTTTGCTTAGTCTTTGCT) (with the *Apa*I site in boldface and the *Xma*I site underlined) and cloned into the plasmid **p**TubH7-HApac to generate the **p**TubRab11-HApac episomal vector.

### 2.2. GlVps4a and GlRab11 Downregulation

For VPS4a antisense, the nucleotide 1-1035 of *glvps4a* open reading frame (ORF) was amplified using the f4 (5′- CAAT**GATATC**CCATTGTTCCTGGTCGAAACATTG-3’) and r3 (5-′ CAATCCATGGCTCATCTGCTAGCACATTGGGCGT-3´) oligonucleotides (with the *EcoR*V site in boldface and the *Nco*I site underlined), restricted and ligated to the **p**TubVps4a-HApac (in the opposite direction), resulting in the antisense vector **p**TubVps4a:aspac, which was used for inhibition of VPS4a expression. To knock down GlRab11, nucleotides 1–639 from ORF of *glrab11* were introduced into the double-stranded RNA vector [44,45], following PCR amplification using the dsRNA gRab11pF 5′-CAAT**GGATCC**ACTGACGCGTACGACCATCTTTA-3′ and dsRNA gRab11pR (a′) 5′-CATTGCATGCGCAACGCTTCTTTTGCTTAGTCTTTGCT-3′ oligonucleotides (with the *BamH*I site in boldface and the *Sph*I site underlined). The correct sequences of all constructs were verified by sequencing.

### 2.3. Giardia Isolates and Transfection

*G. lamblia* trophozoites of the isolate WB, clone 1267 [46], were cultured in TYI-S-33 medium supplemented with 10% adult bovine serum and 0.5 mg/ml bovine bile, as previously described [47]. These trophozoites were used as hosts for the expression of transgenic genes and as non-transfected controls. Trophozoites were transfected with the constructs by electroporation and selected by puromycin as previously described [45,48]. Drug-resistant *glvps4a-ha, glvps4a_E228Q_-ha, glrab11–ha*, *glvps4a:as*, and *ds-glrab11* transgenic trophozoites were usually apparent by 7–10 days post-transfection; 10 μg of the resulting **p**dsRNA-glrab11 vector was used to transfect the *Giardia* clone WB1267 as described above. To induce GlRab11 dsRNA, tetracycline (Tet) (10 μg/ml) was added to *ds-glrab11-*transfected trophozoites [45]. GlRab11p depletion was confirmed by qRT-PCR before performing the analysis of cell growth.

### 2.4. Exosome Enrichment

Exosomes were collected using differential ultracentrifugation from wild-type, *glvps4a-ha*, *glvps4a_E228Q_-ha*, *glvps4a:as*, *glrab11–ha*, or *ds-glrab11* trophozoites. Briefly, 14 × 10^7^ trophozoites recovered from the monolayer were washed twice with warm PBS 1X (37 °C) and incubated with TYI-S-33 medium without serum or bovine bile (TYI-S-33/-sbb) for 4 h. Then, the supernatant was recovered and the parasites were removed by centrifugation at 1455× *g* for 15 min. Cellular proliferation/viability measured after 4 h incubation was determined by counting with a hemocytometer after addition of 0.4% Trypan blue (SIGMA, MilliporeSigma, St. Louis, MO, USA), according to the manufacturer’s instructions. After centrifugation, the supernatant was filtered through a 0.11-µm filter (Millipore) to discard microvesicles. To obtain the exosomal fraction, the filtered supernatant was subsequently pelleted at 100,000× *g* for 200 min using a 60Ti rotor (*Beckman-coulter* L-70 Ultracentrifuge). The pellet was then washed with PBS and pelleted again at 100,000× *g* in the same ultracentrifuge [6]. Finally, the pellet was suspended in 100 µL of PBS and the exosomes were used immediately for sucrose gradient flotation as described [49]. Exosomal pellet was mixed with 300 µl ml of 2.5 M sucrose in PBS and placed on the bottom of 0.5 ml of 2.5 M sucrose (20 mM Tris-HCl, pH 7.4) underlying 0.75 ml of 1 M sucrose. The cushions were ultracentrifuged (TLV.100 rotor) at 100,000× *g* for 15 hours. Cushion fractions were collected from the top of the tube. F1 to F8 (top to bottom) fractions were washed with PBS and pelleting at 200,000× *g* for 1 h using a TLV.100 rotor rotor (*Beckman-coulter* Optima Max-XP Ultracentrifuge). Fresh pellets were subjected to negative staining, electron microscopy, and size determination, as described below.

### 2.5. Negative Staining and Electron Microscopy

Fresh exosomes were diluted in PBS, placed on copper grids, and incubated 15 min at room temperature. Liquid excess was removed by blotting. The grid was placed on 2% uranyl acetate (w/v) (Merck, Darmstadt, Germany) for 30 s and observed in a JEOL 1230 transmission electron microscope.

### 2.6. Dynamic Light Scattering (DLS)

Fresh exosomes were suspended in 100 µL of PBS, and size distribution was measured by DLS using a submicron particle sizer, Nicomp™380 (Particle Sizing Systems, Santa Barbara, California, USA). All measurements were in duplicate at 25 °C. Data processing and analysis was performed using Nicomp software CW380, version 1.51. Each sample was measured in triplicate. 

### 2.7. Immunoblot Analysis

Immunoblot assays were performed as previously reported [50]. Briefly, trophozoites and EVs fractions were suspended in 50 µL of PBS-1%Triton X-100 supplemented with protease-inhibitor mixture (P8340, Sigma) and phosphatase-inhibitor mixture (P2850, Sigma) and set on ice for 1 h. The sample was centrifuged at 24,000× *g* for 30 min at 4 °C, and the supernatant was collected. The protein concentration was measured with the Bradford method (Pierce), and the material was denatured in Laemmli buffer containing 10× Dithiothreitol and was boiled for 10 min. After cooling, the proteins were separated on NuPAGE 4-12% Bis-Tris gel (Thermo Fisher Scientific) in Buffer MOPS 1X. Samples were transferred to nitrocellulose membranes, blocked with 5% skimmed milk and 0.1% Tween 20 in 1X Tris-buffered saline TBS, and then incubated with primary antibody diluted in TBS buffer containing 2.5% skimmed milk and 0.1% Tween 20. After washing and incubation with an enzyme-conjugated secondary antibody, proteins were visualized with the SuperSignal West Pico Chemiluminescent Substrate (Pierce, Thermo Fisher Scientific Inc., Rockford, IL, USA) and autoradiography. The following antibodies were used: anti-giardial 14-3-3 (g14-3-3) rabbit polyclonal serum (pAb) [51], anti-giardial thioredoxin reductase (gTrxR) mouse pAb [52], and mouse anti-giardial Protein disulfide isomerase-2 (PDI-2) [20]. The pAb anti-actin was a gift from Alex Paredez [53]. The anti-gQa1 monoclonal antibody (mAb) (clone 5B2) was a gift from Theodore Nash (unpublished). The anti-alpha tubulin (clone B-5-1-2) was purchased from SIGMA. Protein loading gels are provided in Supplementary Figure S1A.

### 2.8. RT-qPCR

Trizol reagent (Invitrogen, Life Technologies, Carlsbad, USA) was used for the extraction of total RNA from wild-type and transgenic trophozoites, according to the manufacturer’s protocol. SV Total RNA Isolation System (Promega Corporation, Madison, WI, USA) was used for a second RNA purification, according to the manufacturer’s protocol. cDNA synthesis was performed with RevertAid™ Reverse Transcriptase (Fermentas, Massachusetts, USA). cDNA was analyzed for wild-type and transgenic trophozoite genes using real-time PCR SYBR Green Master Mix from Invitrogen (Invitrogen, Life Technologies, Carlsbad, USA), and single stranded cDNA (100 ng of the input total RNA equivalent), and 800 nM of amplification primer were used in a reaction volume of 20 μl. The oligonucleotides GlVps4apFw 5´CGGCGCTGAGAAGAAAGACT-3´, GlVps4apRv 5´-CGCAACAGGCTACTCACAGT-3´, GlRab11pFw 5´-TCTCGAGGTTCACCAGCAAC-3´, and GlRab11pRv 5´-AAGCTCCGTCAACCACTCTG-3´ were used to determine the expression of sense mRNA of Vps4a and Rab11, respectively. The housekeeping gene 18s (f: 5´-AAGACCGCCTCTGTCAATAA-3´ and r: 5´-GTTTACGGCCGGGAATACG-3´ oligonucleotides) was used to normalize the gene expression and was calculated using the ΔΔCt method. The oligonucleotides were designed using Primer Express software (Applied Biosystems, USA). Runs were performed on a 7500 standard system (Applied Biosystems, USA). The relative-quantitative RT-PCR conditions were 50 °C for 2 min, 95 °C 10 min and 40 cycles at 95 °C for 15 s, and 60 °C for 1 min. RT-PCR was performed using the One-step RT-PCR kit (Qiagen, Valencia, CA), as previously described [54]. For DNA contamination control (negative control), corresponding oligonucleotides were added at the PCR step of the RT-PCR reaction. Statistical analysis was carried out using GraphPad Prism software (GraphPad Software, Inc.). The t-test was used to determine differences between wild-type cells (control group) and *glvps4a-ha, glvps4a_E228Q_-ha, glrab11–ha*, *glvps4a:as*, and *ds-glrab11* transgenic cells.

### 2.9. Immunofluorescence Assay (IFA)

IFA assays were performed as described [55]. Briefly, trophozoites cultured in growth medium were harvested and attached to slides, pretreated with poly-L-lysine. The cells were fixed with 4% formaldehyde, washed, and blocked with PBS containing 10% (*v/v*) normal goat serum and 0.1% Triton X-100. Cells were fixed and incubated with the primary mouse anti-HA mAb (clone GT423) (SIGMA) diluted in PBS containing 3% (*v/v*) normal goat serum and 0.1% Triton X-100, followed by incubation with Alexa 488 anti-mouse (dilution 1:500) secondary antibody at 37 °C. Finally, the preparations were washed with PBS and mounted in FluorSave™ mounting medium (Merck, Kenilworth, NJ, USA). Fluorescence staining was visualized by confocal laser scanning microscopy (CLSM) using a motorized FV1000 Olympus microscope (Olympus UK Ltd, UK), with 63x or 100x oil immersion objectives (NA 1.32). Differential interference contrast images were collected simultaneously with the fluorescence images by the use of a transmitted light detector. Images were processed using Fiji software [56].

### 2.10. Transmission Electron Microscopy (TEM) of Giardia trophozoites

Briefly, 14 × 10^7^ wild-type trophozoites, *glvps4a-ha*, *glvps4a:as*, *glvps4a_E228Q_-ha*, *glrab11–ha*, or *ds-glrab11* transgenic trophozoites were grown in axenic culture at 37 °C in 8-ml tubes containing TYI-S-33 medium until monolayer and washed twice with warm PBS 1X (37 °C) and adherent cells fixed with a solution containing 2.5% glutaraldehyde, 4% paraformaldehyde, and sucrose 4% in 0.1 M cacodylate buffer. Then, the cells were scraped off the tube wall with a rubber policeman; washed in 0.1 M cacodylate buffer; and postfixed for 60 min at room temperature in a solution containing 1% osmium tetroxide, 0.8% potassium ferrocyanide, and 5 mM CaCl_2_ in 0.1 M cacodylate buffer. Afterward, the cells were washed in buffer, dehydrated in acetone, and embedded in Polybed resin. Thin sections were stained with uranyl acetate and lead citrate and observed in a JEOL 1230 Transmission Electron Microscope.

### 2.11. Labeling Membranes with Fluorescent Bodipy FL C5-Ceramide

Bodipy FL C5-ceramide (Molecular Probes, Invitrogen) was used to detect ceramide membrane enrichment, as previously performed [57]. Briefly, wild-type trophozoites were suspended in labeling buffer (50 mM glucose, 10 mM cysteine, and 2 mM ascorbic acid in PBS, pH 7.1) containing 5 μg of the fluorescent analog, incubated at 4 °C for 30 minutes, washed, and incubated in slides at 37 °C for 5 h or 9 h. For photoconversion of diaminobenzidine (DAB), the cells were fixed with 4% formaldehyde solution for 40 minutes at room temperature, washed 3 times with 0.1 M sodium cacodylate buffer (pH 7.4), and incubated in a cold solution of 1.5 mg/mL DAB in 0.1 M sodium cacodylate buffer for 15 minutes in the dark. The samples were then irradiated with an argon-ion laser for 30 minutes using a ×10 objective. After irradiation, a brown staining was observed, indicative of a DAB reaction. Finally, samples were processed for TEM as described above.

## 3. Results

### 3.1. G. lamblia Secretes Exosome-Like Vesicles

**Figure 2:**
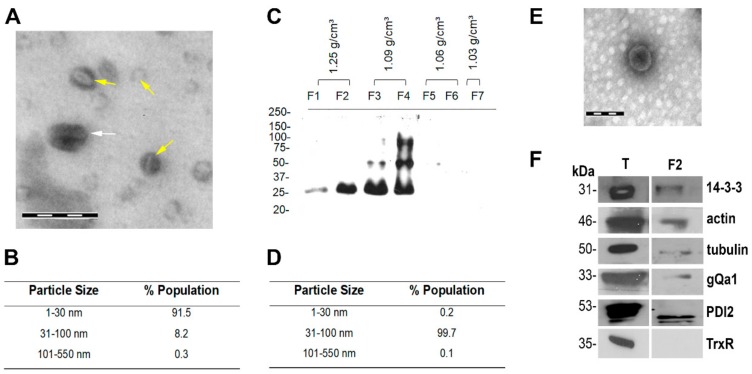
Characterization of exosomal-like vesicles in *Giardia* WB1267 trophozoites: (**A**) Examination of enriched vesicles contrasted with uranyl-acetate by TEM shows vesicles with an exosomal cup-shape appearance of around 70 nm and vesicles (white arrow) of around 20–25 nm (yellow arrows). Bar: 100 nm. (**B**) Table of dynamic light scattering (DLS) trace of vesicles shows the presence of 22.8–103.2 nm and smaller 1.0–22.8 nm vesicles. Average measured values are shown. (**C**) Immunoblot analysis of sucrose gradient fractions shows an enrichment of 14-3-3 in F2, F3, and F4 fractions. (**D**) Table of DLS analysis shows a peak of vesicles of diameters 10.5–73.6 nm in F2. Average measured values are shown. (**E**) Negative staining electron microscopy shows enrichment of cup-shaped vesicles of around 80 nm in F2. Bar: 100 nm. (**F**) Ten μg of total proteins were loaded in 4–12% SDS-PAGE gel to perform immunoblot analysis. The wild-type trophozoites WB1267 homogenate (T) and the exosomal-enriched fraction (F2) tested positive for 14-3-3, actin, tubulin, gQa1, and PDI2. The cytoplasmic enzyme TrxR was used as a negative control for the F2 fraction. Protein molecular weights (kDa) are reported on the left.

Several methods have been published to purify and characterize MVs in the range of 30–150 nm, thus including exosomes [58]. Here, we adapted a classical purification method based on differential ultracentrifugation [6] and modified the *Giardia* culture medium to prevent contamination with exosomes from other sources (i.e., bovine serum and bile). For this purpose, monolayers of *G. lamblia* trophozoites, isolate WB-1267 (Assemblage A), were washed and incubated for an additional 4 hours in TYI-S-33/-sbb medium prior to exosome isolation. After filtration and ultracentrifugation (Supplementary Figure S1B), the pellet was examined by negative staining and TEM. The results showed a population of vesicles with different sizes and shapes, including a subgroup with an exosome cup-like shape and a diameter of 50–100 nm (Figure 2A), which we termed exosome-like vesicles (ElV), together with abundant smaller vesicles (approx. 20–25 nm). Dynamic light scattering (DLS) confirmed the presence of two main populations of vesicles with average sizes of 22.8 and 85.2 nm, with the first population representing the majority of the particles (Figure 2B). To characterize the 50–100 nm vesicles, an additional purification step using a continuous 1.03–1.25 g/cm^3^ sucrose gradient was performed [6,49] and fractions from the gradient were collected and analyzed. Fractions were tested for the presence of giardial 14-3-3 (GL50803_6430), a protein known to be present in exosomes of different origins [59]. Immunoblot analysis of sucrose gradient fractions (F1–F7) showed an enrichment of giardial 14-3-3 at densities ranging from 1.09 to 1.25 g/cm^3^ (Figure 2C), similar to the densities at which the exosomal fraction have been described (1.15-1.19 g/cm^3^) [60]. DSL was performed in all fractions, showing that F2 was the fraction enriched in the 50–100 nm vesicles (Figure 2D and Supplementary Table S1). An exclusive enrichment of 80-nm cup-shaped vesicles was confirmed in this fraction by negative staining and TEM (Figure 2E). DSL and TEM analysis prompted us to select the F2 fraction for further characterization. For this, several giardial homologs of other known “exosome markers” were tested by immunoblotting [61]. In particular, we analyzed the presence of actin (GL50803_40817), alpha-tubulin (GL50803_103676), gQa1 (GL50803_7309), a SNARE (N-ethylmaleimide-sensitive factor attachment protein receptor) associated with the PVs [62,63], and the ER-chaperone protein disulfide isomerase 2 (PDI2) (GL50803_9413) [64]. The “exosome markers”, 14-3-3, actin, and alpha-tubulin were detectable in F2 together with the vacuolar SNARE gQa1 and PDI2 (Figure 2F). The cytosolic enzyme, thioredoxin reductase (TrxR), was found excluded from the ElV fraction F2. These results suggest that the 50–100 nm vesicles represent true exosomes based on their size, shape, and protein markers. Additionally, the presence in these vesicles of the PV-membrane associated SNARE gQa1 points to a PV origin of the ElVs, although the occurrence of the ER-membrane chaperone PDI-2 in the enriched fraction F2 suggests a possible crosstalk between the endoplasmic reticulum (ER) and PVs.

### 3.2. The Giardial GlVPS4a Protein is Involved in ElV and ILV Biogenesis

Considerable data supports the participation of ESCRT machinery in exosome biogenesis during the process of endosomal maturation by being involved in ILV and MVB generation [65]. Indeed, an RNA interference screen targeting 23 proteins of the endosomal ESCRT machinery showed that selected ESCRT components and accessory proteins have a critical role in exosome secretion [8]. In particular, the expression of SKD1/VPS4B, an ESCRT-associated AAA+-ATPase, was found to be relevant for exosome biogenesis in HEK293 cells [66]. Moreover, it was shown that the expression of an ATP hydrolysis-deficient Vps4 (VPS4A-E228Q and VPS4B-E235Q) mutant affected MVB formation [67,68] and EV biogenesis [66] in mammalian cells. Besides the reduced set of ESCRT subunits, recent data show that the remaining subunits present in the *Giardia* genome are expressed in trophozoites and cysts and that at least GlVps25, GlVps2, GlVps4a, GlVps4b, and GlVps4c are functional in yeast, thus arguing in favor of a functional ESCRT machinery in this parasite [22,23]. To investigate the role of the ESCRT complex in exosomal vesicle biogenesis in *G. lamblia*, we analyzed the participation of the AAA+-ATPase GlVps4a (GlVps4a) using the transgenic trophozoites *glvps4a-ha* and *glvps4a_E228Q_-ha* overexpressing GlVps4a-HA or the ATP hydrolysis-deficient mutant GlVps4a E228Q (GlVps4a_E228Q_-HA), respectively. The GlVps4a_E228Q_-HA, similar to other AAA+ ATPase-deficient mutants, likely bind to normal substrates of Vps4 interfering with ESCRT-dependent processes [68,69]. Also, we produced stable *glvps4a:as* transgenic cells in which GlVps4a expression was inhibited by a specific antisense RNA.

**Figure 3:**
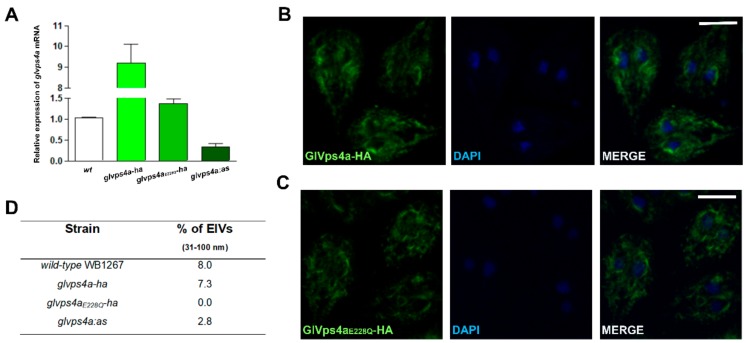
Exosomal release in wild-type and GlVps4a transgenic trophozoites: (**A**) Real-time RT–PCR analysis of GlVps4a gene expression in trophozoites from wild-type WB1267 (wt) and the transgenic *glvps4a-ha*, *glvps4a_E228Q_-ha*, and *glvps4a:as* trophozoites. The assay was performed using primers specific for the *glvps4a* gene. Transcript levels were normalized to *gdh* RNA levels. Average and standard deviation of three independent experiments are plotted. (**B**) Immunofluorescence Assay (IFA) and confocal microscopy of *glvps4a-ha* cells show the reticular localization of GlVPS4a-HA in green using anti-HA mAb and Alexa 488 anti-mouse. Image merging and 4′,6-diamidino-2-fenilindol (DAPI) staining of the nuclei (blue) are shown. Bars: 5 µm. (**C**) IFA and confocal microscopy of *glvps4a_E228Q_-ha* cells show GlVps4a_E228Q_-HA in green using anti-HA mAb and Alexa 488 anti-mouse. Image merging and DAPI staining of the nuclei (blue) are shown. Bars: 5 µm. (**D**) Table of DLS assay showing ElVs production and vesicle size variation. Average measured values are shown.

An increase in the mRNA expression of *glvps4a* was measured in both *glvps4a-ha* and *glvps4a_E228Q_-ha* cells, while a three-fold transcriptional downregulation was achieved in *glvps4a:as* transgenic trophozoites (Figure 3A). Localization of HA-tagged GlVps4a proteins by CLSM analysis showed that both GlVps4a-HA and GlVps4a_E228Q_-HA have a reticular as well as a cytoplasmic localization (Figure 3B,C). We determined the involvement of GlVps4a in ElV biogenesis by quantifying the number of vesicles produced in wild-type and transgenic trophozoites. For this, the number of cells was adjusted and the ElV was recovered from the supernatant after 4 h incubation in the TYI-S-33/-sbb medium by filtration and ultracentrifugation. No difference in cell growth was observed between the transgenic and wild-type trophozoites during the 4 hours of incubation in the TYI-S-33/-sbb medium (not shown). We observed a comparable percentage of vesicles of 31–100 nm diameter in *wild-type* and *glvps4a-ha* but a reduction in ElV production in the knockdown *glvps4a:as* cells (Figure 3D). Interestingly, *glvps4a_E228Q_-ha* cells did not produce ElV, indicating a direct association between GlVps4a inactivation/downregulation and the production of ElVs in *G. lamblia* (Figure 3D). The strong dominant-negative phenotype produced by the overexpression of the GlVps4a_E228Q_-HA is consistent with complete inhibition of the assembled GlVps4a oligomeric structure.

**Figure 4:**
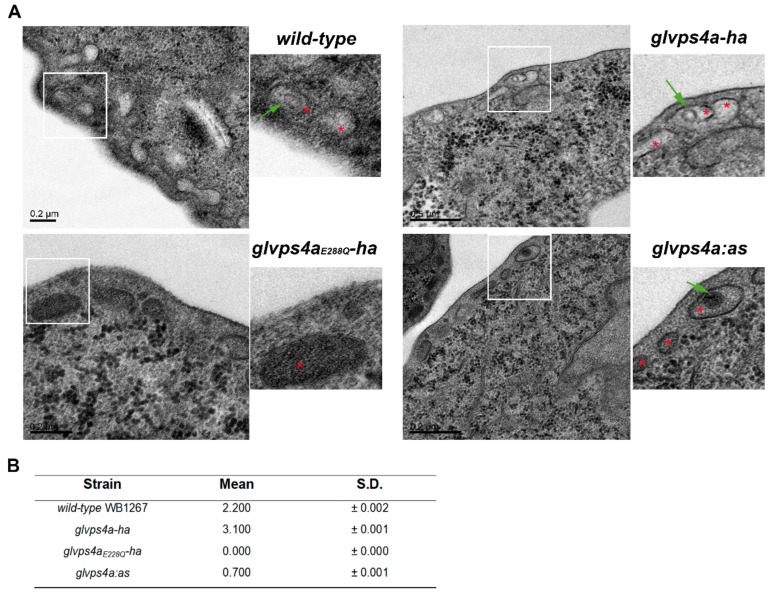
ILVs production in wild-type and GlVps4a transgenic trophozoites: (**A**) TEM of wild-table *WB1267* and the transgenic *glvps4a-ha*, *glvps4a_E228Q_-ha*, and *glvps4a:as* trophozoites showed the presence of ILVs (green arrow in insets) inside the PVs (red asterisk in insets). Bar length is indicated on each picture. (**B**) Table showing the percentage of PVs containing ILVs for each strain. n = 200 per strain (mean +/- SEM).

The possible participation of GlVps4a in ElV production prompted us to analyze whether GlVPS4a (and thus the giardial ESCRT machinery) participated in the genesis of the ILVs in *Giardia*. For this, TEM was performed in fixed wild-type, *glvps4a-ha*, *glvps4a_E228Q_-ha,* and *glvps4a:as* cells, and the percentage of PVs containing at least one ILV was analyzed. The results showed a reduction of ILV genesis in *glvps4a:*as trophozoites and a lack of PVs containing ILVs in the *glvps4a_E228Q_-ha* trophozoites compared to wild-type and *glvps4a-ha* cells (Figure 4A,B). These results are consistent with the marked reduction on ElV generation for *glvps4a:*as and the *glvps4a_E228Q_-ha* trophozoites, thus suggesting that GlVps4a function is critical for exosome production in *Giardia*.

### 3.3. GlRab11 is Necessary for Exosome-Like Vesicle Production

**Figure 5:**
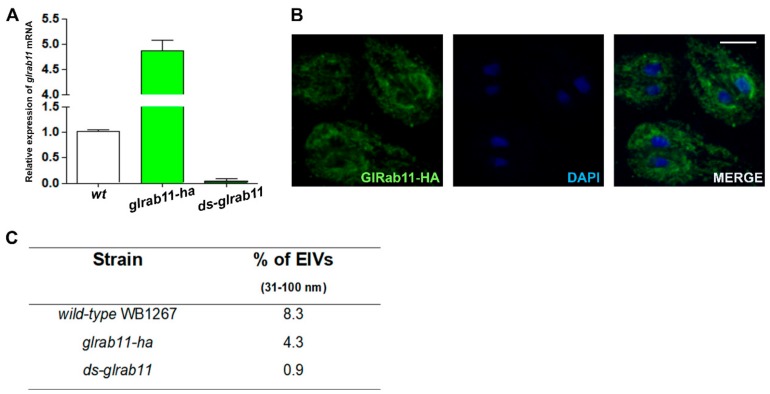
Exosomal release in wild-type and GlRab11 transgenic trophozoites: (**A**) Real-time RT–PCR analysis of GlRab11 gene expression in trophozoites from wild-type WB1267 (wt) and transgenic *glrab11-ha* and *ds-glrab11* trophozoites. The assay was performed using primers specific for the *glrab11* gene. Transcript levels were normalized to *gdh* RNA levels. Average and standard deviation of three independent experiments are plotted. (**B**) IFA and confocal microscopy of *glrab11-ha* cells show GlRab11-HA in green using anti-HA mAb and Alexa 488 anti-mouse. Image merging and DAPI staining of the nuclei (blue) are shown. Bar: 5 µm. (**C**) Table of DLS assay shows ElVs production and vesicle size variation for each strain. Average measured values are shown.

In *G. lamblia*, GlRab11 participates in early and late encystation stages [36] and it was shown to be essential for cytokinesis [35]. In metazoans, Rab11 has been associated with vesicular trafficking to and from recycling endosomes to the plasma membrane and with exosome release [37,38,70]. To understand the involvement of GlRab11 in ElV secretion, we produced cells overexpressing GlRab11-HA *(glrab11-ha)* and double-stranded mRNA of GlRab11 (*ds-glrab11)*. The results obtained by qRT-PCR analysis showed a marked transcriptional upregulation of GlRab11-HA in *glrab11-ha,* while a reduction of GlRab11 mRNA was achieved in *ds-glrab11* (Figure 5A). IFA and confocal microscopy analysis indicated that GlRab11-HA has a reticular and peripheral subcellular localization in log phase *glrab11-ha* transgenic trophozoites (Figure 5B), consistent with a previous report [37]. The recovery of vesicles from the supernatant of wild-type and transgenic cells was performed as described before, and the ElV secretion was analyzed by DLS. No difference in cell growth was observed between the transgenic and wild-type trophozoites during the 4 hours of incubation in the TYI-S-33/-sbb medium (not shown). The results indicated that overexpression of GlRab11-HA increased the release of 31–100 nm diameter vesicles in trophozoites, while GlRab11 downregulation inhibited this process (Figure 5C).

**Figure 6:**
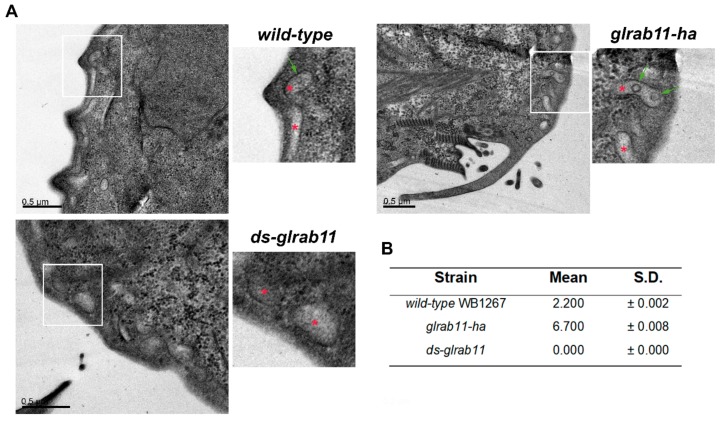
ILVs production in wild-type and GlRab11 transgenic trophozoites: (**A**) TEM of wild-type WB1267 and the transgenic *glrab11-ha* and *ds-glrab11* trophozoites showed the presence of ILVs (green arrow in inset) inside the PVs (red asterisk in inset). Bar length is indicated on each picture. (**B**) Table showing the percentage of PVs containing ILVs for each strain. n = 200 per strain (mean +/- SEM).

To analyze whether GlRab11 is involved in ILV biogenesis, we determined the capacity of transgenic cells to form ILVs by TEM (Figure 6A). The number of PVs containing ILVs was quantified for each case, indicating that the biogenesis of ILVs was dependent on Rab11 (Figure 6B). Interestingly, some PVs showed two ILVs only in *glrab11-ha* transgenic trophozoites. Altogether, these results suggest that GlRab11 participates in ILV biogenesis and ElV production in *G. lamblia*.

### 3.4. External Ceramide Triggers ILVs Budding Formation

As mentioned before, it was shown that ceramide was enriched in the exosomes [10] and that exogenous sphingomyelinase treatment or external addition of C6-ceramide induced the formation of ILVs and exosomes enriched in ceramide in different cell types [71,72,73]. Bodipy-FL C5-ceramide, a marker of the Golgi apparatus in mammalian cells [74,75], was added to analyze the trafficking between membranes of intracellular organelles and exosomes of basophil leukemia cells (RBL-2H3), showing that Bodipy-ceramide fluorescence was 10 times higher in released exosomes than in RBL cells [76].

**Figure 7:**
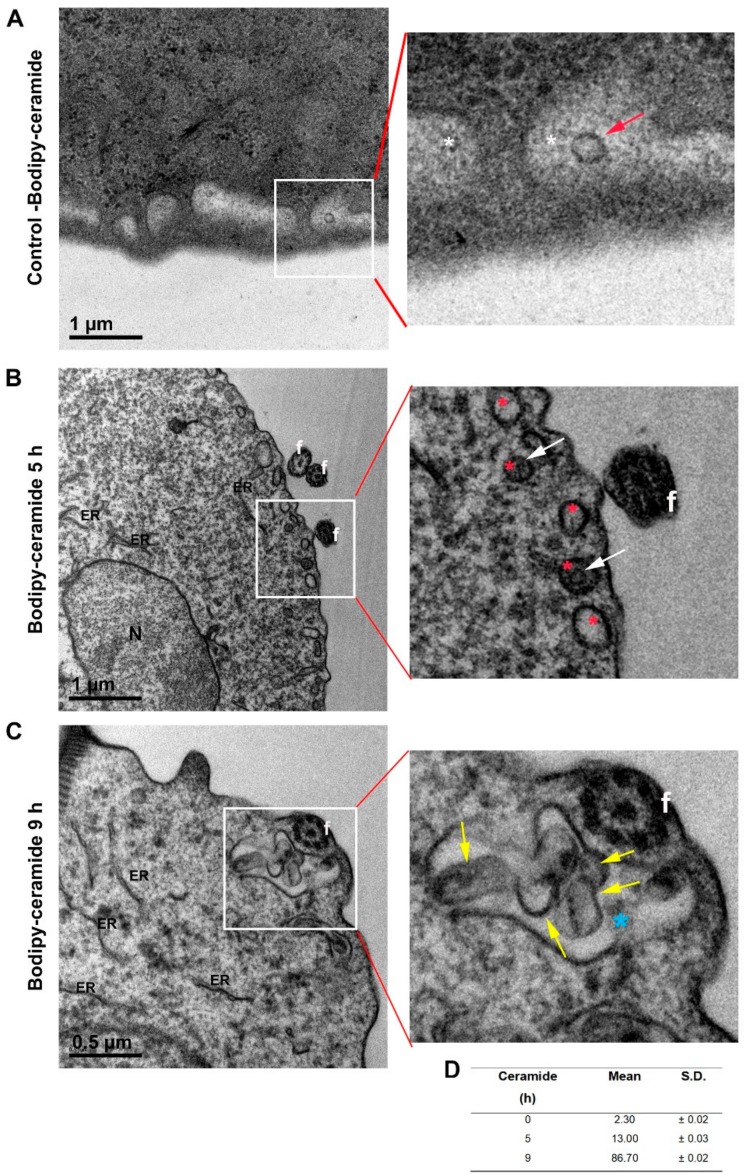
Ceramide content on ILV and PV membranes: (**A**) TEM of negative control without Bodipy FL C5-ceramide. *G. lamblia* PVs containing ILVs are shown. Panel on the right shows in detail one ILV (red arrow) inside the PV (white asterisk). (**B**) TEM localization of Bodipy FL C5-ceramide after diaminobenzidine (DAB) photooxidation shows the black staining of the ER, ILV, and PV membranes (5 h ceramide incubation). Panel on the right shows in detail the dark stain in the ILV membrane (white arrow) inside the PV (red asterisk). (**C**) When the trophozoites were incubated with Bodipy FL C5-ceramide for 9 h, many ceramide-enriched ILVs (yellow arrow) are observed inside a bigger PV (blue asterisk) by TEM. Bar length is indicated on each picture. ER: endoplasmic reticulum. f: flagella. N: nucleus. (**D**) Table showing the percentage of PVs containing ILVs for each treatment. n = 100 per strain (mean +/- SEM).

To determine the role of ceramide in ILV formation in *Giardia*, we evaluated the incorporation of Bodipy FL C5-ceramide to giardial ILVs. We performed diaminobenzidine (DAB) photooxidation for conversion of fluorescent signals from Bodipy FL C5-ceramide into electron-dense precipitates visible by TEM, as previously reported [57]. Trophozoites were labeled with Bodipy-ceramide, and the samples were analyzed by TEM after photooxidation. The results showed that, besides the ER [57], the PVs and the ILV membranes were clearly stained with oxidized DAB, suggesting that giardial ILVs contain ceramide (Figure 7A,B). Moreover, longer external addition of ceramide showed that some PVs contained several ILVs (Figure 7C). The number of PVs with ILVs was quantified for each treatment (Figure 7D). Although these results match recent reports showing that ceramide contributes to the budding of ceramide-enriched intraluminal vesicles, inducing membrane curvature [10], additional experiments are needed to fully characterize the role of ceramide in ILV and exosome biogenesis in this parasite.

## 4. Discussion

Many recent studies highlight the importance of EVs released by protozoan parasites in intercellular communication, including parasite–parasite and parasite–host interactions. The presence of EVs in *G. lamblia* trophozoites and encysting cells has been recently addressed, demonstrating that their secretion is stimulated by the addition of external calcium and that increasing the MVs produces the activation of immature dendritic cells in vitro [5]. In an attempt to provide an exhaustive description of *Giardia* secreted proteins (secretome) of the reference isolates WB and GS [77], the authors reported the release of two types of vesicles (100–250 nm and 100 nm) in the supernatants of trophozoite growth in axenic conditions, in which exosomes may be included. When the *Giardia* secretome was in contact with IEC cells, it resulted in the alteration of IEC gene expression, cell signaling, and the production of proinflammatory cytokines [77]. These authors suggest an immunomodulatory effect of *Giardia* on host immune responses [77], also supported by the observation that *Giardia*’s secretome was able to improve or reduce mast cell protease activity, thereby modulating mast cell-induced immune responses [77]. Interestingly, the proteomic analysis of the secretome of supernatant of axenic WB isolate culture revealed the presence of the protein 14-3-3, tubulins, and PDIs, in agreement with our results. However, much specific information regarding exosome-like vesicles may be lost in these proteomic approaches because of the sample acquisition and exosome representativeness.

In this work, we showed that *Giardia* produces vesicles with exosome characteristics in terms of size, shape, density, and protein markers. These results were obtained after minimizing the source of exosome contamination that may come from the culture medium (serum and bile) and by performing filtration and ultracentrifugation protocols followed by sucrose gradient separation. These analyses also showed that, despite lacking typical MVBs, exosome-like vesicles may be formed in the PVs, since proteins associated with the PV membranes, like the gQa1 and the encystation-specific cysteine protease (not shown) [63], were enriched in the exosomal fractions obtained from axenic cultures of growing WB trophozoites. Interestingly, the presence of PDIs in the exosomal fraction suggests a participation of the ER in ILV formation, in coincidence with the reports that the ER and PVs are somehow interconnected [20]. This observation differs from other reports that suggest that exosomes lack protein components of intracellular compartments out of the endosomal pathway [78].

The discovery that ESCRT machinery is involved in the biogenesis of MVBs enriched the new field of exosome biogenesis. However, the current opinion is that there are at least three independent mechanisms responsible for the classification of exosomal proteins: ESCRT mediated, lipid mediated, and tetraspanin mediated. It seems that these mechanisms coexist and are responsible for the classification of different proteins and/or many different vesicle subpopulations. In an evolutionary analysis of the ESCRT components and the presence of MVBs in eukaryotic lineages, Field et al. showed that, although the genus *Giardia* has an extreme evolutionary divergence and often results in the lack of recovery of ortholog candidates, this does not explain the complete failure in the detection of sequences encoding some ESCRT proteins [79]. Instead, they suggest that this parasite contains a minimal ESCRT/MVB machinery, probably as a result of an unusual endocytic system [79].

The absence of ESCRT components in *G. lamblia* correlates with the lack of genes encoding ESCRT-I and -II; and several subunits of ESCRT-III; and associated complexes in *P. falciparum*, *Toxoplasma gondii*, and *Cryptosporidium parvum* [79]. However, a significant difference between *G. lamblia* and these organisms is that they have organelles morphologically similar to MVBs [80,81]. In this context, a recent publication suggesting that PVs containing ILV resemble atypical MVBs supports the hypothesis that PVs may be the only components of the endo-lysosomal system in this parasite [82]. In search of the participation of the ESCRT machinery in the biogenesis of ILVs and ElVs in *Giardia* trophozoites, we found that transgenic cells overexpressing the ATP hydrolysis-deficient mutant GlVps4a_E228Q_ or the antisense of *glvps4a* mRNA showed a reduction of ILV formation on the PVs, which was correlated with the absence of ElV release when compared to wild-type cells. Moreover, transgenic trophozoites overexpressing GlVps4a-HA showed more ILVs, although the production of ElVs was similar to that of the wild-type, suggesting that there is another related mechanism involved with exosome release that is independent of GlVps4a. In this sense, the Rab proteins have been associated with MVB docking and exosome release in different cell types, including Rab2, Rab7, Rab11, Rab27a/b, and Rab35 [33,41,70,83,84,85]. However, the specific role of the Rab proteins in exosome biogenesis and release seems to be species specific, showing significant differences. When we analyzed the participation of GlRab11 in ElV production in *Giardia*, we found that it was critical, since GlRab11 downregulation inhibited ElVs and ElV genesis in Rab11-lacking transgenic cells. Conversely, overexpression of GlRab11 raised the number of PVs containing ILVs but not ElV release, suggesting that GlRab11 participates also in the biogenesis of ILVs rather than in ElV discharge. This agrees with the role of Rab11 in other organisms in which it has been associated with trans-Golgi network membranes and secretory vesicles besides its function in the exocytic pathway [9,86]. Since *Giardia* lacks a morphologically discernible Golgi apparatus, the reticular and peripheral localization of GlRab11 suggest that it may play a role in ER–PV communication rather than in PV docking to the plasma membrane. The trafficking between the ER and the PVs in *Giardia* has been addressed, showing that vesicular trafficking was possible between these two organelles [45,87,88]. Moreover, the ER marker glucose 6-phosphatase was also observed in the PVs while the soluble lysosomal acid phosphatase, a PV marker, was found in the ER as well as in the lumen of the PVs [17,89]. Supporting the ER–PV interaction, Abodeely et al. (2009) [20] suggested that the tubular structure of the giardial ER has points of contact with the PVs, but other reports showed that the ER membranes do not invade the PV space [26,90]. Still, all the evidence points to a close communication between the ER and the PVs.

Beside the proteins, lipids are a crucial molecule for ILVs and exosome formation and the analysis of the lipid composition may help to understand their biological role. Ceramide enrichment was shown in the membranes of ILV and exosomal fractions in other organisms [10]. One proposal is that ceramide may induce membrane curvature, facilitating the formation of ILVs regardless of the protein composition (like ESCRTs and tetraspanin) [11,12]. Our results revealed that exogenous ceramide localized in the ER, PV, and ILV membranes, showing that, as in other cells, ceramide is a component of the ILVs in this parasite. Moreover, the addition of exogenous ceramide increased ILV formation inside the PVs. These results are an advance toward a complete lipidomic screening of the ElVs in *Giardia* that may also help to conclude that differential and independent exosome biogenesis exists in *Giardia*.

## 5. Conclusions

One of the most interesting scientific findings of biological significance was that extracellular vesicle (EVs) release is not waste disposal of the cell but, rather, a complex system of intercellular communication. Despite great efforts during recent decades in this field, we must recognize that there are many more questions than answers, in part, because of limitations in the EV separation and analysis techniques. However, one way to overcome the problems is by associating the size, form, and composition of each EV with their biological functions. Thus, our work starts to shed light on the mechanism governing the release of exosome-like vesicles (ElVs) in the highly reduced model organism of *G. lamblia* and links their formation with the unusual participation of the incomplete ESCRT complex and Rab11. Our findings show the involvement of conserved players (ESCRT, Rab, ceramide, etc.) in ElV biogenesis but also indicate some particularities, such as the crosstalk between the ER and the PVs, supporting the data that EVs carriers are species specific. This work paves the way for further studies to elucidate the functions of ElVs in *G. lamblia* by the characterization of their content (proteins, lipids, and nucleic acids) in multi-omics studies and to evaluate whether ElVs may play a role in parasite–parasite communication.

## Supplementary Materials

The following are available online at https://www.mdpi.com/2073-4409/8/12/1600/s1, Figure S1: (A) Schematic view of the isolation methods employed to enrich exosomes from Giardia culture. (B) Protein profile from trophozoite homogenate (T) and F2 fraction., Table S1: Size of EVs as determined by DLS
