# Supplementary material for: Exosome Biogenesis in the Protozoa Parasite Giardia lamblia: A Model of Reduced Interorganellar Crosstalk
Source: Cells. 2019 Dec 9;8(12):1600. doi: 10.3390/cells8121600 (PMC6953089; doi:10.3390/cells8121600)
Supplement: Supplementary file 1 [file cells-08-01600-s001.pdf]

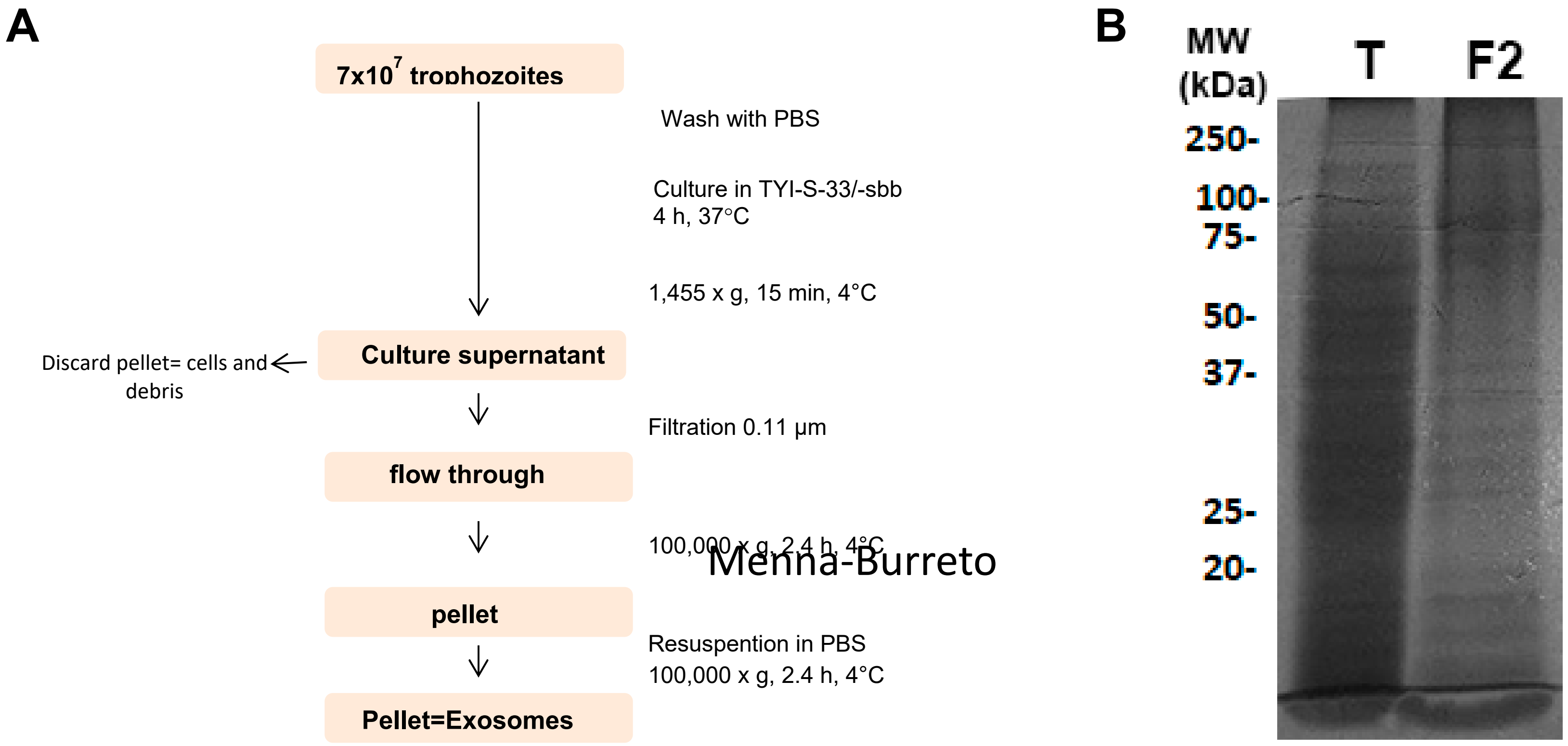

Figure S1. (A) Schematic view of the isolation methods employed to enrich exosomes from *Giardia* culture. (B) Protein profile from trophozoite homogenate (T) and F2 fraction. An equivalent amount (10 µg) of each sample was loaded, run on a NuPAGE 4-12% Bis-Tris gel and stained with Coomassie blue. MW markers are shown on the left.

Table S1: Size of EVs as determined by DLS

Fraction	Particle size	% Population
F1	1-30 nm	100.0
	31-100 nm	0.0
	101-550 nm	0.0
F2	1-30 nm	0.2
	31-100 nm	99.7
	101-550 nm	0.1
F3	1-30 nm	97.7
	31-100 nm	2.3
	101-550 nm	0.0
F4	1-30 nm	83.0
	31-100 nm	16.5
	101-550 nm	0.5
F5	1-30 nm	99.5
	31-100 nm	0.5
	101-550 nm	0.0
F6	1-30 nm	97.9
	31-100 nm	2.1
	101-550 nm	0.0
F7	1-30 nm	99.8
	31-100 nm	0.2
	101-550 nm	0.0
